# Cytogenetic Characterization of Seven Novel satDNA Markers in Two Species of Spined Loaches (*Cobitis*) and Their Clonal Hybrids

**DOI:** 10.3390/genes11060617

**Published:** 2020-06-04

**Authors:** Anatolie Marta, Dmitry Dedukh, Oldřich Bartoš, Zuzana Majtánová, Karel Janko

**Affiliations:** 1 Laboratory of Fish Genetics, Institute of Animal Physiology and Genetics, Czech Academy of Sciences, 27721 Liběchov, Czech Republic; didukh@iapg.cas.cz (D.D.); bartos@iapg.cas.cz (O.B.); majtanova@iapg.cas.cz (Z.M.); janko@iapg.cas.cz (K.J.); 2 Department of Zoology, Faculty of Science, Charles University in Prague, 128 00 Prague, Czech Republic; 3 Institute of Zoology, Academy of Science of Moldova, MD-2028, Academiei 1, 2001 Chisinau, Moldova; 4 Department of Biology and Ecology, Faculty of Science, University of Ostrava, Chittussiho 10, 710 00 Ostrava, Czech Republic

**Keywords:** clonal vertebrates, hybridization, satellite DNA, FISH, mitotic and lampbrush chromosomes

## Abstract

Interspecific hybridization is a powerful evolutionary force. However, the investigation of hybrids requires the application of methodologies that provide efficient and indubitable identification of both parental subgenomes in hybrid individuals. Repetitive DNA, and especially the satellite DNA sequences (satDNA), can rapidly diverge even between closely related species, hence providing a useful tool for cytogenetic investigations of hybrids. Recent progress in whole-genome sequencing (WGS) offers unprecedented possibilities for the development of new tools for species determination, including identification of species-specific satDNA markers. In this study, we focused on spined loaches (*Cobitis*, Teleostei), a group of fishes with frequent interspecific hybridization. Using the WGS of one species, *C. elongatoides*, we identified seven satDNA markers, which were mapped by fluorescence in situ hybridization on mitotic and lampbrush chromosomes of *C. elongatoides*, *C. taenia* and their triploid hybrids (*C. elongatoides* × 2*C. taenia*). Two of these markers were chromosome-specific in both species, one had centromeric localization in multiple chromosomes and four had variable patterns between tested species. Our study provided a novel set of cytogenetic markers for *Cobitis* species and demonstrated that NGS-based development of satDNA cytogenetic markers may provide a very efficient and easy tool for the investigation of hybrid genomes, cell ploidy, and karyotype evolution.

## 1. Introduction

Interspecific hybridization is a driving force in evolution and may have various outcomes ranging from the promotion of interspecific barriers to the establishment of successful stable lineages of hybrid origin [1,2,3]. A growing body of evidence suggests that hybridization can lead to the emergence of clonal (so-called “asexual”) lineages which often co-exist with one of the parental species [4,5]. Such a process has been intensively studied especially in teleost fishes, including the genera *Cobitis*, *Carassius*, *Misgurnus*, *Squalius*, and *Chrosomus* [6,7,8,9,10,11,12].

A particularly well-studied case of hybridization-induced clonality encompasses the spined loaches of the genus *Cobitis*; small-sized, bottom-dwelling fishes with about 60 species that inhabit shallow freshwaters across Palearctic realm [13]. The Central European *Cobitis* hybrid complex comprises of several parental species and their hybrids. *C. elongatoides* diverged from the common ancestor approx. 9 Mya and closely related species *C. taenia*, *C. tanaitica*, *C. pontica*, and *C. taurica* lineages that reproduce via gynogenesis in the Western Palearctic realm [14,15,16]. In particular, the species pair *C. taenia* (TT) and *C. elongatoides* (EE) served as an excellent model in the investigation of mechanisms controlling the origin and reproduction of hybrid clones [3,9]. However, as in most other asexual taxa, the investigation is hampered by extreme difficulties in the taxon identification owing to the relative morphological similarity between the parental species and their hybrids, which occur in several forms that differ in genomic composition. In particular, the genomic doses from parental species may be equal in some hybrids (i.e., diploids and symmetrical tetraploid—ET, EETT) or may differ as in triploids (ETT, EET) and asymmetrical tetraploids (ETTT, EEET). Moreover, the morphological characteristics of hybrids represent a continuum between the parental species [16,17,18].

Typically, precise genome and/or ploidy identification relied on a combination of multiple diagnostic approaches: sequencing of multiple mitochondrial and nuclear loci, allozyme, and cytogenetic analyses (e.g., karyotyping and C-banding, rDNA FISH) [9,18,19]. However, many important scientific questions regarding the consequences of hybridization, polyploidy, and asexuality on chromosomal and genome evolution require more fine-scale markers allowing the identification of sub-genomes within the nuclei of hybrids. Until now, identification of European *Cobitis* hybrids and their karyotype composition relied on conventional cytogenetic markers, mainly focusing on the distribution and abundance of rDNA clusters. Recent studies showed that karyotypes of parental species have diverged from a common ancestor by various chromosomal rearrangements involving one possible fusion and numerous pericentric inversions [20]. As for the Central European spined loaches, the karyotype of *C. elongatoides* (2n = 50) possesses the most distinct karyotype dominated by meta- and submetacentric chromosomes, while the karyotype of *C. taenia* (2n = 48) is dominated by acrocentric and subtelocentric chromosomes [20]. However, despite their karyotypic differences, both species can hybridize in nature, and as a result, one would expect a high number of polymorphisms in the wild mixed populations. The lack of consensus on the precise categorization of karyotypes among species caused by the absence of species-specific cytogenetic markers also leads to ambiguous interpretations of results achieved by different research groups [21,22].

Implementation of novel NGS data to cytogenetics currently represents one of the most promising tools for unveiling genome evolution. Comparative analysis of the distribution of repetitive DNA markers within the karyotype by fluorescence in situ hybridization (FISH) appears particularly useful in that context. The availability and affordability of whole-genome sequencing (WGS) represent an important source of data that has the power to help us to shed light on how genetic information is stored and organized in chromosomes and how it has evolved over time. The genome of teleost fishes contains a variety of repetitive DNA sequences that are widely dispersed and typically arranged in tandem repeats, e.g., rDNA, satellites, and telomeric DNA [23]. Such repetitive elements are considered one of the principal factors responsible for differences in genome size [23,24]. Satellite DNA sequences (satDNA) consist of highly repetitive noncoding monomers with an average length of 100 to 700 bp, often organized in large tandem clusters [25]. Satellite DNA may play a structural role in genome organization and it could also influence gene expression [26,27,28]. Often, genetic incompatibilities between genomes of species have been associated with variations in satDNA abundance that are frequently present in the centromeres and telomeres of chromosomes, as well as in heterochromatic regions [24].

The rapid evolution and constant homogenization of the genome or at least of its repetitive content, i.e., “concerted evolution” [29], gives rise to species-specific sequences [30,31]. The molecular drive process plays a role in the evolution of satDNA that becomes fixed within the sexual ancestral population [32] and generates divergence between species (or reproductive groups), leading to the diversification of satDNA [33]. The decreasing costs of WGS and subsequent rise of different bioinformatics tools for de novo satDNA characterization, such as the TAREAN (TAndem REpeat ANalysis) software [34], allowed for the expansion of comparative genomic and cytogenetic studies.

In this study, we performed a genome-wide analysis of *C. elongatiodes*, which allowed us to identify seven major satDNA repeats. We mapped these markers on mitotic metaphase and lampbrush chromosomes (LBC) of *C. taenia*, *C. elongatoides*, and their triploid clonal hybrids ETT. The cytogenetic mapping of these markers using FISH allowed the unambiguous identification of the genomic composition of the hybrids.

## 2. Materials and Methods 

### 2.1. Selection of Specimens and Ethics Statement

In this study, we analyzed six individuals of *C. elongatoides*, five individuals of *C. taenia* and six hybrid ETT specimens from the rearing facility stocks of the Laboratory of Fish Genetics, IAPG CAS in Liběchov that had been collected during former projects across central Europe in accordance with environmental protection legislation. The Valid Animal Use Protocol was in force during the study at the IAPG (No. CZ 02386). All institutional and national guidelines were covered by the “Valid Animal Use Protocol” No. CZ 02386 of the Laboratory of Fish genetics. Taxonomic identification and genotyping of examined individuals were based on previously determined and routinely applied molecular markers involving several allozyme loci, one sanger-sequenced nuclear intron (S7), and one mitochondrial gene (cytochrome b) [9,35]. Total genomic DNA was extracted from muscle tissue using the Dneasy Blood and Tissue Kit (Qiagen) following a standard protocol. The cyt*b* gene was amplified according to [9] and S7 gene following [16]. PCR products were commercially Sanger-sequenced (Macrogen Korea and Europe). Sequences were aligned in ClustalW [36] and manually edited in Mega 7.0. 

### 2.2. Bioinformatics Analyses

We utilized the whole genome sequencing data of *C. elongatoides* generated on the Illumina HiSeq X platform (paired-end 2 × 150, insert size 450 bp). 

As those data equaled roughly 40× coverage of the genome (estimated size ~1.5 gbp), random 500,000 read-pairs were sampled using the seqtk sample tool (available from https://github.com/lh3/seqtk) from both forward and reverse fastq files using the seed to ensure proper pair sampling. This sampling resulted in the final ~0.075× coverage of the genome.

All reads were trimmed by seqtk trimfq from both sides equally to the final length of 100 bp and were converted into the fasta format. Reads were further formatted in R [37] using the package seqinr [38], including read names and overall data format. Finally, fasta files were formatted using the script reformat.sh from the BBtools [39] (out = *.fa tuc = T fastawrap = 105).

The TAREAN [34] software uses low coverage WGS data to predict and reconstruct repetitive elements of a genome. In contrast to k-mer analysis based approaches, e.g., Tandem Repeat Finder [40], TAREAN pipeline uses unassembled reads that are clustered according to their similarity [41], resulting in virtual graphs whose shapes reflect the genomic organization and sequence variability of corresponding repeats, ranging from linear structures typical for dispersed transposable elements to circular graphs of tandemly repeated sequences [34]. Note that such graphical shapes (clustering patterns) are usually not easily resolved by short-read assemblers, which can lead to the underrepresentation of repetitive sequences in genomic assemblies [42]. Therefore, pipelines using non-assembled short reads shall be better suited for such types of analysis, but TAREAN still seems to be the only pipeline that implements such a strategy [43].

### 2.3. Preparation of Mitotic Chromosomes 

Cell suspensions with mitotic chromosomes from all individual specimen were obtained either from kidneys following the protocol of [44] and/or regenerated caudal fins according to [45]. Metaphase chromosomes were initially stained with Giemsa to check the ploidy level and the morphology of chromosomes.

### 2.4. Preparation of Lampbrush Chromosome 

Lampbrush chromosomes (i.e., chromosomes in diplotene stage) were prepared from both parental species females as well as hybrid ones according to [46] with modifications proposed by [47]. Vitellogenetic oocytes with size 0.5–1.5 mm in diameter were taken from hormonally non-stimulated females were resuspended in OR2 saline medium (82.5 mM NaCl, 2.5 mM KCl, 1 mM MgCl_2_, 1 mM CaCl_2_, 1 mM Na_2_HPO_4_, 5 mM HEPES (4-(2-hydroxyethyl)-1-piperazineethanesulfonic acid); pH 7.4). Nuclei were then microsurgically isolated from oocytes by jeweller forceps and needles in the isolation medium “5:1” (83 mM KCl, 17 mM NaCl, 6.5 mM Na_2_HPO_4_, 3.5 mM KH_2_PO_4_, 1 mM MgCl_2_, 1 mM DTT (dithiothreitol); pH 7.0–7.2). Nuclear envelopes were manually removed in one-fourth strength “5:1” medium with the addition of 0.1% paraformaldehyde and 0.01% 1 M MgCl_2_ in a chamber attached to a slide meaning that in each chamber we obtained chromosome spread from individual oocytes. Slide with oocyte nuclei contents were subsequently centrifuged for 20 min at +4 °C, 4000 rpm, fixed for 30 min in 2% paraformaldehyde in 1× PBS, and post-fixed in 70% ethanol overnight (at +4 °C). Description of bivalent morphology and lampbrush chromosome map construction were performed according to [48] in Corel™ DRAW graphics suite X8 software.

### 2.5. DNA Probes and Fluorescent In Situ Hybridization (FISH)

Based on the TAREAN results, seven predicted satellites were selected and uploaded to the NCBI repository with the given accession numbers MT454818–MT454824, and different primers pairs designed for each of them (Table 1). For DNA amplification and probe preparation, gDNA of *C. elongatoides* was used, using commercial kit Qiagen DNeasy Blood & Tissue Kit. Probes were amplified and labeled by PCR. PCR reactions were performed in 1× PCR buffer, 1.5 mM MgCl_2_, 200 µM each dNTP, 0.1 µM each primer, 2 pg–10 ng of gDNA, and 0.5 U of Taq polymerase (all reagents from TopBio, Prague, Czech Republic). The cycling program for amplification consisted of an initial denaturation at 95 °C for 5 min, followed by 30 cycles of 95 °C for 20 s, 63 °C for 30 s, and 72 °C for 20 s and a final extension at 72 °C for 15 min. Probes were labeled with biotin-16-dUTP (Roche Applied Science, Mannheim, Germany) and digoxigenin-11-dUTP (Roche Applied Science, Mannheim, Germany) through PCR reamplification of PCR products, using the same PCR conditions as described above.

For FISH, chromosome slides were incubated with 0.01% pepsin/0.01 M HCl for 10 min, fixed with 2% paraformaldehyde (PFA) for 10 min. Slides with lampbrush chromosomes were not pre-treated before hybridization. Denaturation of probes was performed directly on slides in hybridization mix (50% formamide, 10% dextran sulfate, 2× SSC, 5 ng/μL labeled probe and 10–50-fold excess of tRNA) at 77 °C for 10 min. After hybridization overnight at room temperature (RT) all slides were washed three times in 0.2× SSC for 5 min at 44 °C. Detection of probes was performed with avidin-FITC and anti-digoxigenin-rhodamine (Roche Applied Science, Mannheim, Germany), chromosomes were mounted with Vectashield DAPI antifade medium (Vector Laboratories, Burlingame, CA, USA).

### 2.6. Image Processing

Chromosomal preparations were examined by an Olympus Provis AX 70 epifluorescence microscope and ZEISS Axio Imager.Z2 epifluorescence microscope. Images of metaphase chromosomes were recorded with a cooled Olympus DP30BW CCD camera and a CoolCube 1 camera (MetaSystems, Altlussheim, Germany). The IKAROS and ISIS imaging programs (Metasystems, Altlussheim, Germany) were used to analyze grey-scale images. The captured digital images from FISH experiments were pseudocolored (blue for DAPI, red for anti-digoxigenin-rhodamine, green for streptavidin-FITC) and superimposed using Microimage and Adobe Photoshop software, version CS5, respectively. Image processing and chromosomal maps were made in Corel™ DRAW graphics suite X8 software. Chromosomes were classified according to Levan [49].

## 3. Results

### 3.1. Identification and Mapping of satDNA Repeats

TAREAN analysis generated 41 putative satellites (8 of high confidence and 33 of low confidence) and 4 putative long terminal repeat (LTR) elements. Based on the index of satellite probability provided by the TAREAN, we selected seven candidate repetitive elements with lengths ranging from 128 bp to 1820 bp for subsequent FISH mapping. The selected satellites were annotated as satCE01-satCE07 and they included also one putative LTR element (satCE06); Table 2). All these elements were compared against all GenBank records using BLASTn to check for previous annotations. Nevertheless, no significant matches were found in the Genbank database. The length of selected satDNAs and their genome proportion are summarized in Table 2. The nucleotide sequence for each satellite monomer is presented in Supplementary data. 

### 3.2. Karyotypes 

The karyotype of *C. elongatoides* possessed diploid chromosome number 2n = 50 and the number of chromosome arms, i.e., NF value = 76. The karyotype was composed of 46 meta-/submetacentric (M/SM) + 4 subtelo-/acrocentric (ST/A) chromosomes, of which one pair is heteromorphic [21,50] C. taenia possessed 2n = 48, NF = 76 and karyotype was composed of 28 M/SM + 20 ST/A chromosomes. The results of our karyotype analysis were in full accordance with former studies [20,50].

### 3.3. Mapping of satDNA Markers

FISH mapping of candidate satellites have then been performed on metaphase chromosomes as well as on lampbrush chromosomes to specify the location selected markers. To confirm overlapping or co-localization of selected markers, we performed FISH with all possible combinations of two probes (double FISH) on chromosomes of both species and their triploid hybrid. 

**Figure 1:**
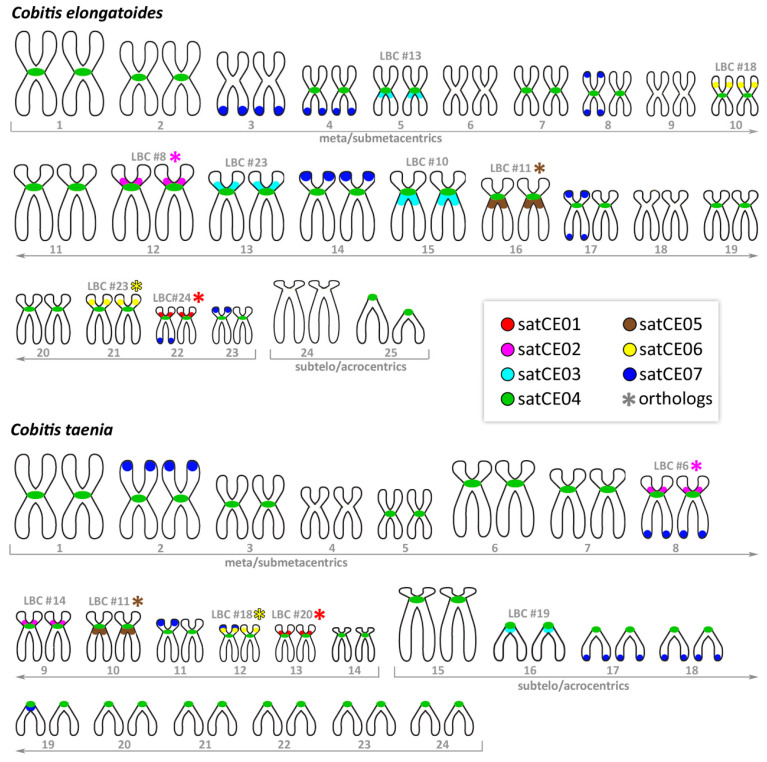
Representative ideograms. Schematic representations of karyotypes of *C. elongatoides* and *C. taenia* showing the distribution of the satDNA markers satCE01–satCE07. An asterisk indicates possible orthologous chromosome pairs between species. The numbers above chromosome pairs indicate respective assignment to lampbrush chromosomes (LBC) based on lampbrush chromosome maps (Supplementary Figure S3). SatCE07 signals are presented on one of the homologs probably due to the absence of equal distribution of this satDNA on homologous chromosomes. Alternatively, it could be that the satCE07 signal on another homologous chromosome is under the discrimination resolution of the FISH method.

**Figure 2:**
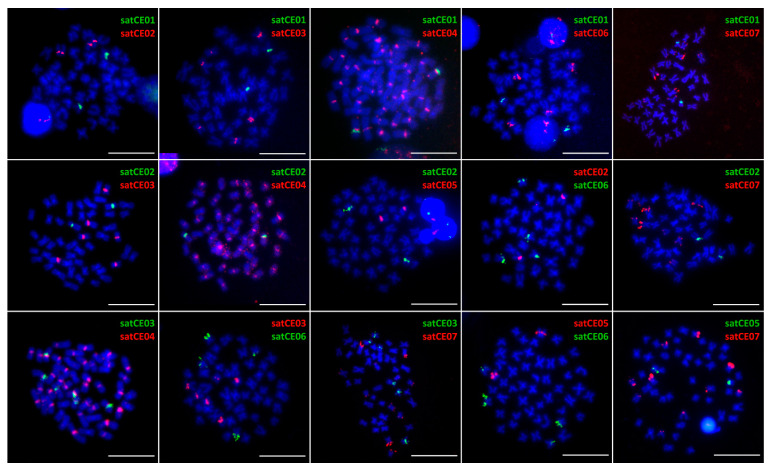
Mapping of satDNA markers on metaphase chromosomes of *C. elongatoides*. Representative mitotic metaphases after double-colored FISH showing positions of satellites satCE01–satCE07 on chromosomes. SatCE02, satCE03, satCE06, and satCE07 show an interspecific polymorphism in the number of detected signals in *C. elongatoides*. Bars equal 10 µM.

**Figure 3:**
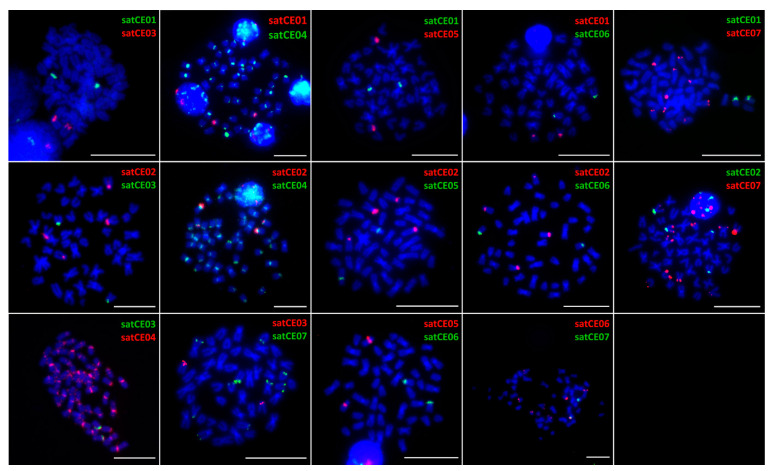
Mapping of satDNA markers on chromosomes of *C. taenia*. Representative metaphases after double-colored FISH showing the position of satellites satCE01–satCE07 on chromosomes. SatCE02, satCE03, satCE06, and satCE07 show an interspecific polymorphism in the number of detected signals in *C. taenia*. Bars equal 10 µM.

Results of all FISH experiments are summarized in representative ideograms demonstrating the physical positions of markers on C. elongatoides and C. taenia metaphase chromosomes (Figure 1). We observed various types of hybridization signals for identified markers. Two markers were chromosome-specific (satCE01 and satCE05), one had centromeric location (satCE04) and four satDNA markers (satCE02, satCE03, satCE06, and satCE07) had variable distribution patterns between examined genomes. Even though SatCE04 was located in the centromeric region likely in all chromosomes of karyotypes of both species (Figure 1, Figure 2, and Figure 3), the signals were not detected on four chromosome pairs of *C. elongatoides*, and one pair of *C. taenia* chromosomes. The intensity of signals was variable and most likely depended on satDNA cluster size on each chromosome. SatCE07 displayed numerous FISH signals scattered on 8 to 10 chromosomes. It was represented by dots in terminal regions, without interstitial sites on chromosome arms. Double-colored FISH experiments with the SatCE07 probes in combination with the other six markers allowed us to identify a potential polymorphism between homologous chromosomes. For example in *C. elongatoides*, the satCE01 probe hybridized on one homologous chromosomal pair, while the Sat07 co-located with only a single chromosome from this pair (Figure 1 and Figure 2). In *C. taenia*, asymmetric co-localization of this marker was detected on chromosomes with probe for satCE06 (Figure 1 and Figure 3). In ETT hybrids the number of signals for each marker corresponded to the combination of the haploid genome of *C. elongatoides* and 2n genome of *C. taenia* (Table 2, Supplementary Figure S1). Signals of satDNA after FISH experiments were clearly visible also in germ cells (Figure S2). This could be used as a tool for the identification of ploidy level.

**Figure 4:**
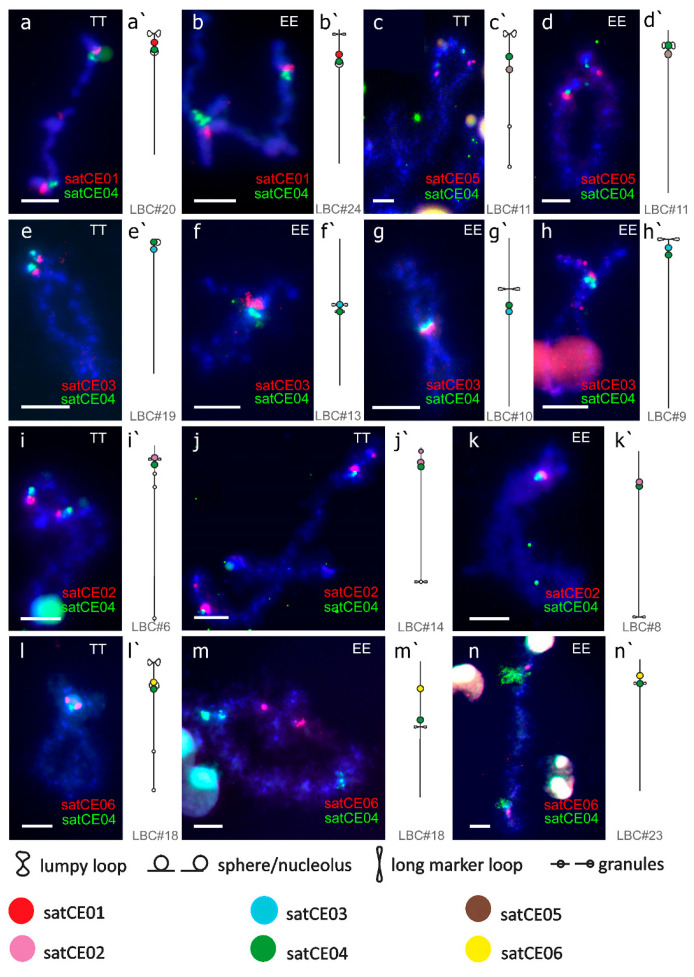
Analyses of lampbrush chromosomes of *C. elongatoides* and *C. taenia*. High-resolution mapping of selected markers on lampbrush chromosomes forming bivalents during diplotene phase of meiotic division and corresponded lampbrush chromosomal maps. satCE04 marker (**green**) indicates centromere position on all presented lampbrush chromosomes of *C. elongatoides* (EE) and, *C. taenia* (TT). satCE01 repeat (**red**) was localized on lampbrush chromosomes no. 24 in *C. elongatoides* (**a**,**a’**) and no. 20 (**b**,**b’**) in C. taenia. satCE05 repeat (**red**) was mapped on lampbrush chromosome no. 11 in both *C. elongatoides* (**c**,**c’**) and *C. taenia* (**d**,**d’**). satCE03 repeat was localized on lampbrush chromosome no. 19 (**e**,**e’**) in *C. taenia* and on 3 lampbrush chromosomes in *C. elongatoides*: no. 9 (**f**,**f’**), no. 10 (**g**,**g’**), and no. 13 (**h**,**h’**). satCE02 marker located on lampbrush chromosomes no.6 (**i**,**i’**) and no. 14 (**j**,**j’**) in *C. taenia* and lampbrush chromosome no. 8 (**k**,**k’**) in *C. elongatoides*. satCE06 marker was mapped on lampbrush chromosome no.18 (**l**,**l’**) in *C. taenia* and lampbrush chromosomes no. 18 (**m**,**m’**) and no. 23 (**n**,**n’**) in *C. elongatoides* Bar equals 5 µM.

For description of satDNA signals on lampbrush chromosomes, we used previously published maps of lampbrush chromosomes [48] (Figure 4). High-resolution mapping of satDNA markers on lampbrush chromosomes from diplotene oocytes allowed for the detailed mapping of each repeat and confirmed the results of previous FISH experiments on metaphase chromosomes. Moreover, we were able to observe more detailed characteristics of satDNAs, e.g., the presence of two clusters of satCE02 on a bivalent annotated as No. 14 in *C. taenia* (Figure 4j). The location of satDNA markers on lampbrush chromosomes can indicate the orthology of respective chromosomes between both species, despite the differences in size and centromeric index. For example, we propose the orthology of bivalent No. 24 in *C. elongatoides* and No. 20 in *C. taenia* based on FISH with satCE01. The identification of orthologous bivalents based on analyses of lampbrush chromosomes are shown in Supplementary Figure S3. Similarly to analyses of metaphase chromosomes, FISH-based mapping of satCE04 allowed to identify centromeric regions of almost all lampbrush chromosomes in *C. taenia* and *C. elongatoides* (Figure 4). Although we detected the satCE07 marker on lampbrush chromosomes of both species, its weak signal and unstable location did not allow us to map it unambiguously. Interestingly, observations of lampbrush chromosomes under phase-contrast optics revealed visible centromeres on bivalents of *C. taenia*, but not on *C. elongatoides*. A similar observation was described by [48] who mapped centromeres in *C. taenia* as granules on lampbrush chromosomes. Together with the precise description, detection of hybridization signals of satDNA on lateral loops of lampbrush chromosomes serves as reliable cytological evidence of DNA transcription. We visualized transcripts of centromeric repeats on several bivalents (Figure 4n).

## 4. Discussion

### 4.1. Karyotype Evolution within Spined loaches of the Central European Hybrid Complex

Teleost fishes show rather conserved karyotypes with a uniform haploid chromosome number n = 24–25 or close to it [51,52], suggesting a slow rate of karyotype changes, which are mostly limited to intra-chromosomal rearrangements [53]. The karyotypes of cypriniform fishes are also relatively conservative (2n = 50), with few exceptions [53] among Cobitoidea and Cyprinidae where cases of polyploidization have been described [3,9,15,18]. Identification of parental chromosomes in hybrids between closely related or nascent species is challenging and requires powerful tools for identification. Such tools can combine next-generation sequencing and classical cytogenetic approaches. Using TAREAN pipeline we developed and mapped seven markers to investigate the karyotypes of two species from the genus *Cobitis* and their triploid hybrids. Our results provide a suitable tool for further investigation of the complex phylogeography, hybridization, and clonality, observed within this group.

Diploid chromosome numbers of species within this group are stable (2n = 50) with the only exception in *C. taenia* (2n = 48) [9,54]. Its karyotype contains a remarkable large metacentric chromosome with a variable size between different populations, which is considered to be the result of the centric fusion of two acrocentric chromosomes [20]. However, the absence of interstitial telomeric signals [22] suggests that they were either lost during the chromosomal rearrangements [55,56], or they are below the detection efficiency of our FISH methodology [57]. A distinct feature in the *C. elongatoides* karyotype is the presence of only one pair of heteromorphic subtelocentric chromosomes [20], while other species of the complex have 5–10 pairs of non-heteromorphic subtelocentric/acrocentric chromosomes. The origin of such heteromorphism could be explained by several hypotheses: 1) *C. elongatoides* could be of a hybrid origin between two closely related species or between individuals from two isolated populations, such an obligatory heterozygous state previously was reported in newts and frogs [58,59]; 2) heteromorphic chromosomes could appear due to inner evolution, e.g., unequal deletions or accumulation of repetitive elements without any impact on meiotic chromosome pairing and formation of bivalents [60,61]. 

Another significant difference between karyotypes of *C. elongatoides* and *C. taenia* is the variability in chromosomal morphology (the number of meta-, submeta-, and acrocentric chromosomes), rDNA sites, NOR sites, and C-banding patterns. Such polymorphism suggests the occurrence of specific chromosomal rearrangements such as inversions, translocations, and fission or fusions, which may have occurred during their evolutionary history [62]. Using our newly designed markers, we did not find any evidence of major translocations or pericentric inversions, probably due to the similar position of studied markers on orthologous chromosomes of *C. taenia* and *C. elongatoides*. Our results indicate that chromosomal evolution within these species might be driven by minor chromosomal changes (e.g., transpositions, insertions, or duplications) because we observed different sizes of orthologous chromosomes, centromeric indexes and variability in bivalents morphology. The chromosomal localization of markers in this study is mostly in pericentromeric regions, suggesting the evolutionary stability of these regions in comparison to subtelomeric and interstitial ones. This observation is in line with satDNA analyses of other vertebrates species [63]. Mapping of satDNAs contributes to the development of genetic markers (chromosomes specific and centromeric markers), which are of significant importance for fundamental and applied biology in fish species [23].

### 4.2. Interspecific Variability of Satellite DNAs 

Satellite DNA represents a dynamic part of heterochromatin, usually being localized in centromeric and subcentromeric regions of chromosomes [25]. For an example of mammals, reptiles, insects, and plants was shown the major role of satDNA in the dynamic evolution of genomic architecture. Furthermore, satDNA together with the flanking regions of these sequences are considered to function as “hotspots” for structural chromosomal rearrangements, contributing to the generation of key variations in the genomes of closely and distantly related species [64]. It is known that satDNA families are often retained in related species during long evolutionary periods [65,66]. Although we expected to identify and validate new species/specific markers, all markers identified by the present study were detected in both species. Hence, in accordance with the “library hypothesis” [67], they seem to have originated from a common ancestor of all *Cobitis sensu stricto* loaches.

The abundance of repetitive sequences is very dynamic and can be drastically changed due to expansions and contractions of satDNA arrays, leading to significant differences of satDNA copy numbers among related species [64]. Accordingly, we found that *C. taenia* differs from *C. elongatiodes* in satDNA distribution and cluster size in 5 analyzed markers. In particular, satCE02 repetitive DNA marker was detected on two pairs of chromosomes in *C. taenia*, while in *C. elongatiodes* only one pair shows a clear signal. SatCE03 and satCE06 provided opposite patterns with the presence on 3 and 2 pairs of chromosomes in *C. elongatoides*, respectively, while in *C. taenia* both satDNA elements were detected only in one pair. SatCE04 is a centromeric marker, being present in nearly all chromosomes except one pair of chromosomes in *C. taenia* and 5 pairs of *C. elongatoides*. Centromeric satDNA repeats are highly variable among species, therefore these could serve as a species-specific marker, known for the hybridogenetic *Pelophylax* complex [68] or clonally reproducing *Misgurnus* loaches [69]. On the other hand, in Tilapiinae, several related species share the same centromeric satellite family [70]. We have observed a similar pattern within *C. elongatoides* and *C. taenia*, with the presence of satCE04 in both species. However, the cluster size of this repeat differed between species, suggesting variable copy numbers of repetitive sequences. 

A distinctive feature of the lampbrush chromosomes is the intensive transcription of satellite repeats that leads to formation of extended lateral loops emerging from chromomeres [71,72]. Our study reports for the first time in fish the active transcription of the centromeric satDNA (Figure 4m, satCE4). So far, transcripts of pericentromeric satDNA were reported in amphibian and avian lampbrush chromosomes [25] and also in actively proliferating somatic cells of various animals including mammals [73]. The phenomenon of satellite DNA transcription in lampbrush chromosomes of vertebrates can be hypothetically explained by its regulatory role of maternal non-coding RNA in the early stages of embryogenesis, reviewed in [72,74].

SatDNAs located in subtelomeric chromosomal regions are one of the most rapidly evolving fractions of eukaryotes [75,76]. The only satDNA marker with subtelomeric location, SatCE07, is highly polymorphic and shows a scattered pattern through the genome with 8–10 signals in both parental species. Moreover, we repeatedly observed a signal on a single homolog from a given chromosomal pair. Such an observation could be explained by the absence of equal distribution of this satDNA on homologous chromosomes as a result of chromosomal interaction during meiosis [77,78,79]. An alternative explanation could be that the satCE7 signal on another homologous chromosome is under the resolution of the FISH method. It is also considered that subtelomeric satellite DNA may buffer terminal genes against the processes of loss and gain at the chromosomal ends, although probably without any crucial role in telomere function [80].

We successfully applied our markers on chromosomes of hybrid individuals and found them suitable to unambiguously identify their genomic composition. Earlier, cytological identification of species from this complex comprised only of the description of chromosomal morphology and rDNA markers (5S rDNA, 28S rDNA) [19]. However, rDNA markers in *C. taenia*, are highly polymorphic even within different individuals from the same population [22,81], making them an insufficient tool to observe evolutionary traits or unambiguously infer the genomic composition of hybrids. In this study, we found species polymorphic markers in *C. taenia* and *C. elongatoides* (satCE02, satCE03, satCE06) which allowed us to distinguish parental chromosomes within hybrid karyotypes. Moreover, the application of chromosome-specific markers can serve as a powerful tool to detect ploidy within hybrids of this complex even on interphase nuclei. Our results suggest the chromosomal stability of triploid hybrids with clonal reproduction because the mapping of satDNA on chromosomes shows no changes in the numbers of signals of the analyzed satDNA in comparison to parental species. Even though clonal hybrid lineages were established at least 25,000 years ago [82], such a period of time seems to be insufficient for the accumulation of significant changes in the studied satDNA markers. Our results thus confirm the previous observations of the absence of large-scale chromosomal rearrangements within the Central European *Cobitis* hybrid complex [20,48].

## 5. Conclusions

In this study, we developed seven novel satDNA markers and characterized their distribution and abundance in two species and their triploid asexual hybrids belonging to the Central European *Cobitis* hybrid complex. Among them, we describe both highly conserved, i.e. with a strict chromosome-specific distribution, (satCE01, satCE05) as well as polymorphic (satCE02, satCE03, satCE04, satCE06, and satCE07) markers. We mapped these markers on lampbrush chromosomes which gave us a better idea about their size, localization, abundance, and evolutionary origin. Our results thus suggest that developing new satDNA markers by combining NGS data and classical cytogenetic approaches may greatly improve the understanding of genome evolution and dynamics in asexual hybrid complexes.

## Figures and Tables

**Table 1:** Sequences of primers used in this study.

Satellite	Forward Primer 5′-3′	Reverse Primer 5′-3′
SatCE01	TTTGGGGCAGTCTTGTTGGT	CGTGTGCCCATAGCTCTTCA
SatCE02	ACAGTGTGGTTGGCAGTTGA	GCTGGGAACTAGATGCTTGGT
SatCE03	TCACCCCTGTCCTGTACCAA	ACCTGATCCGGCACAGAAAG
SatCE04	GCTCAGAGCAGCGTTTTACA	ACATCTGCATGTTGCTGTGAAC
SatCE05	AAAGGACCTGT ACGTTGGGC	ACCTTTGAGCAGGGTCTTCG
SatCE06	CCCTGCTCGTCCTACATGAAC	CGGGTGAAAAAGGCAATGGG
SatCE07	GCCACCTCAGGTCAATCTCC	CGAAAGCAG GGGTTTGCTTC

**Table 2:** Characteristics of satDNA markers and their presence on chromosomes of *Cobitis* species.

Satellite	Length (bp)	Genome Proportion *	# of Signals in *C. elongatoides*	# of Signals in *C. taenia*	# of Signals in *C. elongatoides* × 2*C. taenia*
SatCE01	1010	0.07%	2 SM	2 SM	3 SM
SatCE02	910	0.11%	2 SM	4 SM	5 SM
SatCE03	1400	0.079%	6 M	2 A	5 (3 M + 2 A)
SatCE04	128	0.6%	40–42	46	~67
SatCE05	628	0.049%	2 SM	2 SM	3 SM
SatCE06	1820	0.077%	4 (2 M + 2 SM)	2 SM	4 (1 M + 3 SM)
SatCE07	607	0.017%	8–10	8–10	12–15

* The estimated genome proportion of individual satellites according to the TAREAN pipeline [34].

## Supplementary Materials

The following are available online at https://www.mdpi.com/2073-4425/11/6/617/s1. Figure S1: Mapping of satDNA markers on chromosomes of triploid hybrids *C. elongatoides* × 2*C. taenia*; Figure S2: satCE01 detection in mitotic chromosomes (a) and germ cells (b) of *C. elongatoides*; Figure S3: Maps of lampbrush chromosomes of *C. elongatoides* and *C. taenia*.
