# Supplementary material for: Cytogenetic Characterization of Seven Novel satDNA Markers in Two Species of Spined Loaches (Cobitis) and Their Clonal Hybrids
Source: Genes (Basel). 2020 Jun 4;11(6):617. doi: 10.3390/genes11060617 (PMC7348982; doi:10.3390/genes11060617)
Supplement: Supplementary file 1 [file genes-11-00617-s001.pdf]

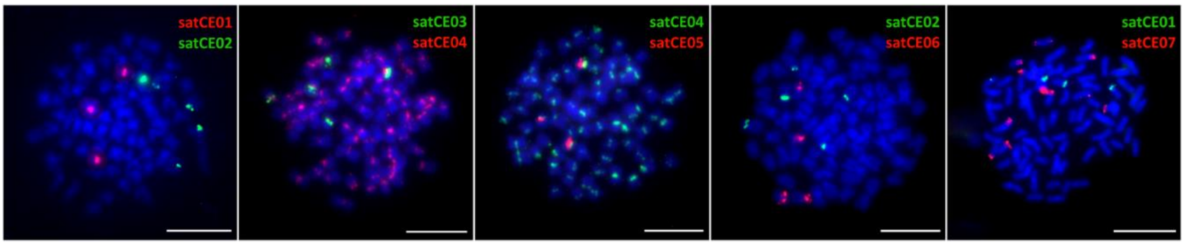

Figure S1. Mapping of satDNA markers on chromosomes of triploid hybrids *C. elongatoides* × 2*C. taenia*. Representative metaphases after double-coloured FISH, indicating mutual position of satCE01 – satCE07. Bars equal 10 μm.

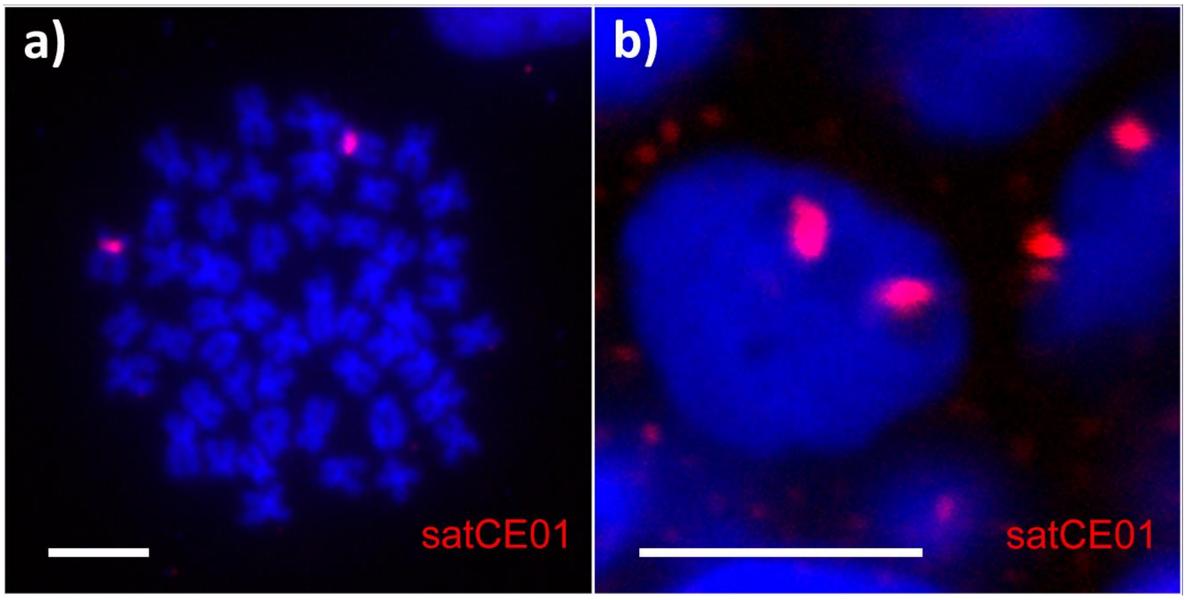

Figure S2. satCE01 detection in mitotic chromosomes (a) and germ cells (b) *of C. elongatoides*. Bars equal 5 μm.

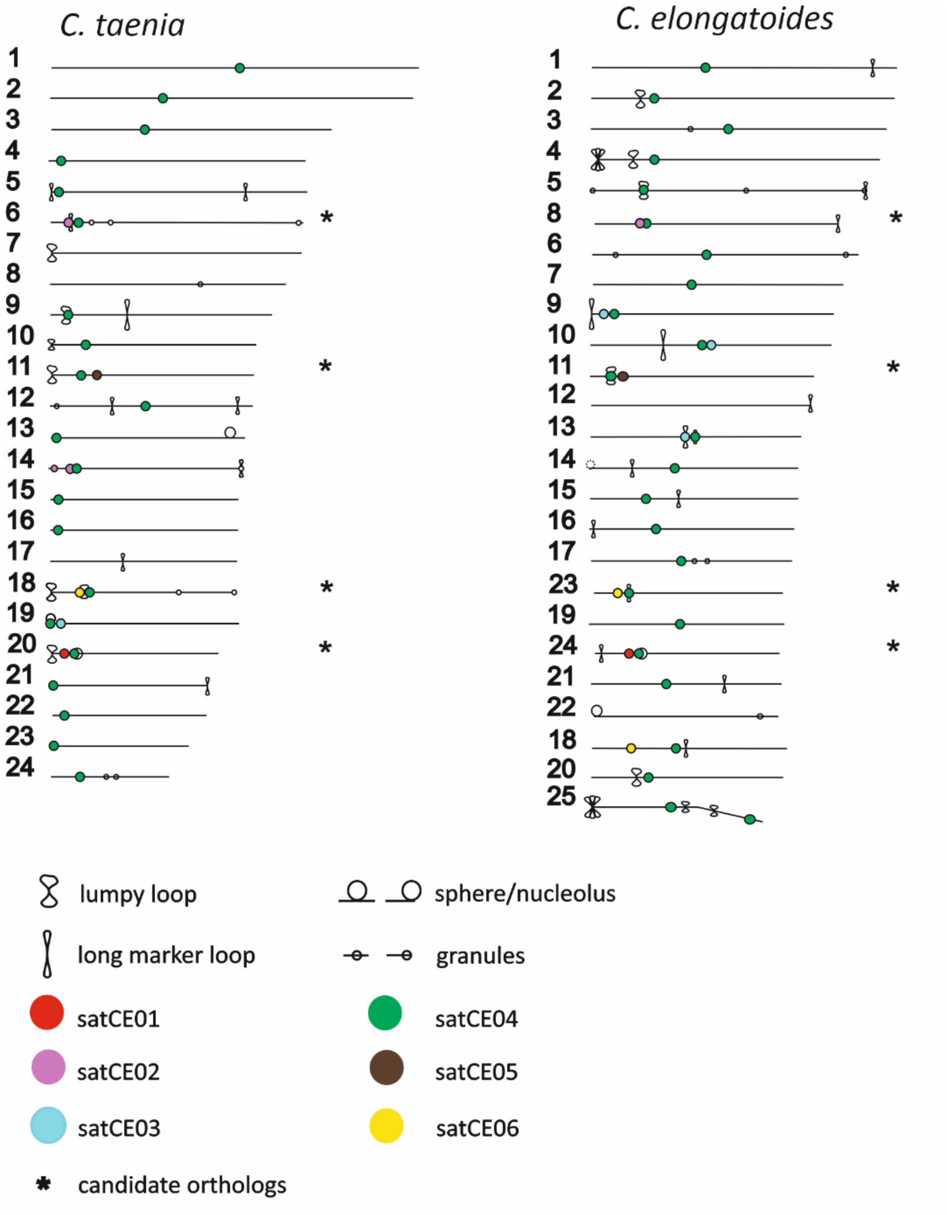

Figure S3. Maps of lampbrush chromosomes of *C. elongatoides* and *C. taenia*. Maps of lampbrush chromosomes with indication of relative position of selected satDNA markers. .
